# Direct oral anticoagulants for oral anticoagulants-naïve Asian patients with atrial fibrillation and end-stage renal disease undergoing dialysis

**DOI:** 10.1038/s41598-024-65541-z

**Published:** 2024-06-25

**Authors:** Jae-Hyung Roh, Yong-Giun Kim, Doyeon Kim, Sangwoo Park, Kyung Sun Park, Minsu Kim, Ki Won Hwang, Wonmook Hwang, Gyung-Min Park, Jae-Hwan Lee

**Affiliations:** 1 Division of Cardiology, Chungnam National University Sejong Hospital, Chungnam National University School of Medicine, Sejong, Korea; 2 Department of Cardiology, Ulsan University Hospital, University of Ulsan College of Medicine, Ulsan, Korea; 3 Graduate School of Data Science, Kyungpook National University, Daegu, Korea; 4 Department of Nephrology, Ulsan University Hospital, University of Ulsan College of Medicine, Ulsan, Korea; 5 Division of Cardiology, Pusan National University Yangsan Hospital, Pusan National University of Medicine, Yangsan, Korea

**Keywords:** Cardiology, Atrial fibrillation, End-stage renal disease

## Abstract

In Asian patients with atrial fibrillation (AF) and end-stage renal disease (ESRD) undergoing dialysis, the use of direct oral anticoagulants (DOACs) remains debatable. From the national health insurance claims data in South Korea, we included 425 new users of OAC among patients with non-valvular AF and ESRD undergoing dialysis between 2013 and 2020. Patients were categorized into DOAC (n = 106) and warfarin group (n = 319). Clinical outcomes, including ischemic stroke, myocardial infarction (MI), intracranial hemorrhage (ICH), and gastrointestinal (GI) bleeding, were compared between the two groups using inverse probability of treatment weighting (IPTW) analysis. During the median follow-up of 3.2 years, the incidence of ischemic stroke was significantly reduced in the DOAC compared to the warfarin group [Hazard ratio (HR) 0.07; P = 0.001]. However, the incidence of MI (HR 1.32; P = 0.41) and GI bleeding (HR 1.78; P = 0.06) were not significantly different between the two groups. No ICH events occurred in the DOAC group, although the incidence rate did not differ significantly between the two groups (P = 0.17). In Asian patients with AF and ESRD undergoing dialysis, DOACs may be associated with a reduced risk of ischemic stroke compared with warfarin. The MI, ICH, and GI bleeding rates may be comparable between DOACs and warfarin.

## Introduction

Atrial fibrillation (AF) is the most commonly treated arrhythmia and is associated with an increased risk of ischemic stroke^1,2^. To prevent ischemic stroke, oral anticoagulants (OACs) are recommended in patients with a high risk of thromboembolism. Direct oral anticoagulants (DOACs) are recommended in preference to warfarin other than patients with mechanical heart valve or moderate to severe mitral stenosis due to their favorable risk–benefit profile with significant reduction in stroke, intracranial hemorrhage (ICH), and mortality and similar bleeding rates compare to warfarin^3,4^.

The prevalence of AF is higher in patients with end-stage renal disease (ESRD) undergoing dialysis than in the general population^5^. Although ESRD is associated with an increased risk of stroke among patients with AF^6^, the safety and benefits of warfarin for ischemic stroke prevention in patients with AF and ESRD undergoing dialysis are questioned^7–9^. In patients undergoing dialysis, DOACs are generally avoided, as their clearance depends on renal excretion. In addition, dialysis patients have been excluded from randomized controlled trials (RCTs) of DOACs^10–13^. Therefore, the efficacy and safety of DOACs in patients with AF and ESRD undergoing dialysis remain uncertain. This study aimed to evaluate the effect of DOACs compared to warfarin on clinical outcomes in Asian patients with AF and ESRD undergoing dialysis.

## Methods

### Data sources

Present study was performed based on the National Health Insurance (NHI) claims data of South Korea from the database of Health Insurance Review and Assessment Service (HIRA)^14,15^. Data were extracted from 2012 to 2021 claims records of the HIRA database. In the HIRA’s claims database, information of patient was de-identified. Diagnoses were coded based on the International Classification of Diseases, Tenth Revision (ICD-10). Additionally, codes from the HIRA database were used to identify about the procedure and medications. This study was approved by the Institutional Review Board (IRB) at Chungnam National University Sejong Hospital, Sejong, South Korea (approval number: CNUSH2022-03-018). As the study subjects were de-identified, the IRB waived the written informed consent. All methods were carried out in accordance with relevant guidelines and regulations.

### Study population

We identified patients aged 20 years and older who were diagnosed with AF undergoing dialysis (hemodialysis or peritoneal dialysis) from the database of the HIRA between September 1, 2013 and August 31, 2020. The date for first diagnosis of AF during the study period was referred to as the index date. To examine patients with ESRD on chronic dialysis, we excluded those with a dialysis period of less than 8 weeks. We also excluded patients who had previous OACs (warfarin or DOACs) prescriptions within 12 months prior to the index date to analyze only those who were new OACs users. Patients with pre-existing mechanical heart valves or mitral stenosis were excluded. Patients with a history of stroke, transient ischemic attack, or ICH were also excluded to focus on the primary prevention of them. Then, patients were divided into DOACs group (dabigatran, rivaroxaban, apixaban, and edoxaban) and warfarin group according to the prescribed OACs within 20 weeks after the index date. We excluded patients who were not prescribed OACs or who were prescribed the OACs less than 90 days.

### Study variables

Comorbidities including hypertension (HTN), diabetes mellitus (DM), dyslipidemia, heart failure (HF), myocardial infarction (MI), systemic arterial embolism, peripheral arterial disease, chronic liver disease, gastrointestinal (GI) bleeding, and unclassified major bleeding were ascertained using the ICD-10 codes. Supplementary Table S1 online summarize the definitions of comorbidities in detail. All prescribed drugs are meticulously and accurately recorded in the HIRA database. Patients were considered to have DM and HTN if anti-diabetic and anti-hypertensive medications were identified from the drugs codes within 12 months prior to the index date^15^. In addition, we identified drugs used, such as beta-blockers, calcium channel blockers, angiotensin-converting enzyme inhibitors (ACEIs)/angiotensin II receptor blockers (ARBs), statins, loop diuretics, proton pump inhibitors, and non-steroidal anti-inflammatory drugs (NSAIDs). The type of OACs [DOACs (dabigatran, rivaroxaban, apixaban, or edoxaban) or warfarin] and the dose of DOACs (standard or reduced) were also identified. In addition, adherence to OACs therapy was evaluated. It was measured as the medication possession ratio (MPR) which is a ratio of the total days’ supply of OACs to the total number of days of study participation per participant^16^. The CHA_2_DS_2_-VASc and modified HAS-BLED scores were calculated by the comorbidities and medications (Supplementary Table S1 online).

### Clinical outcomes and follow-up

Four clinical outcomes were identified to determine the efficacy and safety of DOACs and warfarin: ischemic stroke, MI, ICH, and GI bleeding. Clinical outcomes were defined using the ICD-10 codes (Supplementary Table S1 online). The HIRA database was used in this study to evaluate clinical outcomes through August 31, 2021.

### Statistical analysis

Continuous variables were summarized as the mean ± standard deviation or median (25th, 75th percentiles), and/or categorical variables were summarized as the frequency (percentage). In order to compare categorical data, Chi-square or Fisher’s exact tests were used. The Student’s *t*-test was used to compare continuous variables. The cumulative incidences were estimated using the Kaplan–Meier method with the log-rank test for clinical outcomes. To lessen potential confounding variables in the demographics and comorbidities between the DOAC and warfarin group, this study used the propensity-score method. The propensity-score was assessed using a logistic regression model including variables in the baseline characteristics: age, gender, HTN, DM, dyslipidemia, HF, MI, systemic arterial embolism, peripheral arterial disease, chronic liver disease, GI bleeding, unclassified major bleeding, medications, CHA_2_DS_2_-VASc score, and modified HAS-BLED score. On the basis of calculated propensity-score, inverse probability of treatment weighting (IPTW) was used to balance the covariates between the two groups. The balance of covariates between the two groups was evaluated by their standardized mean differences (SMDs) using a threshold of 0.2 (20%) to indicate imbalance. The weighted incidence rates were calculated as the weighted number of clinical events during the follow-up period divided by 100 person-years at risk. The risk of clinical events was compared between the DOAC and warfarin group via the Cox proportional hazard regression model in the well-balanced cohorts. The proportional hazards assumption was tested on the basis of Schoenfeld residuals. Hazard ratio (HR) and 95% confidence interval (CI) were calculated using the Cox proportional hazard model. Statistical analyses were performed using R software, version 4.2.3 (R Foundation for Statistical Computing, Vienna, Austria). All reported P values were two-sided, and P values of < 0.05 were considered statistical significance.

## Results

### Study population and baseline characteristics

**Figure 1:**
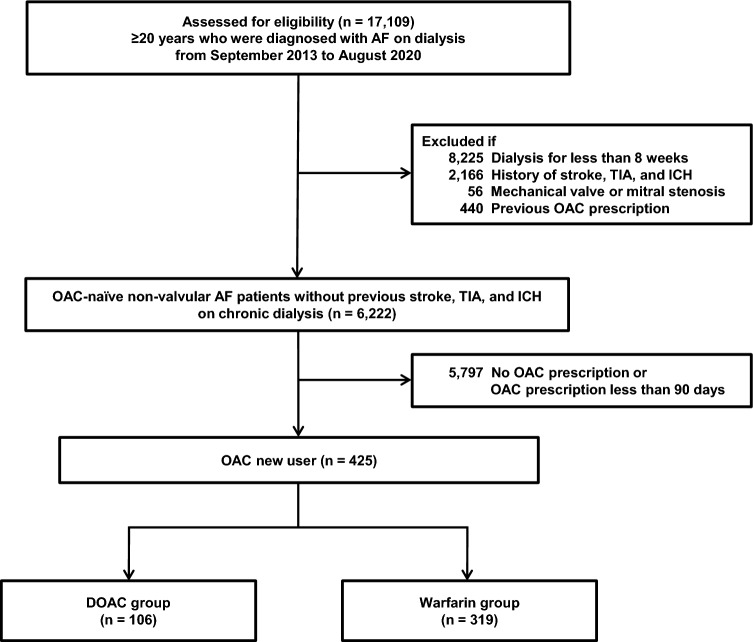
Diagrammatic representation of the study. *AF* atrial fibrillation, *DOAC* direct oral anticoagulant, *ICH* intracranial hemorrhage, *OAC* oral anticoagulant, *TIA* transient ischemic attack.

From September 1, 2013 to August 31, 2020, a total of 17,109 patients aged 20 years and older who were diagnosed with AF on hemodialysis or peritoneal dialysis were identified. Among them, 425 patients met the eligibility criteria and were included in the study (Fig. 1). Most of dialysis was performed via hemodialysis (n = 423, 99.5%). A total of 106 (24.9%) and 319 (75.1%) patients were newly administered DOACs and warfarin, respectively. Table 1 presents the baseline characteristics of the study population according to the type of OAC. The DOAC group had a higher likelihood of DM and dyslipidemia than the warfarin group. In addition, the DOAC group was prescribed more clopidogrel, ACEIs/ARBs, and statins than the warfarin group. There was no difference in the CHA_2_DS_2_-VASc and modified HAS-BLED scores between the two groups. Among DOACs users, apixaban was the most commonly prescribed (72.6%), followed by rivaroxaban (16.0%), edoxaban (8.5%), and dabigatran (2.8%). Most of DOAC users (89.6%) were prescribed a reduced dose of DOAC. The median MPR of the DOAC and warfarin group were 72.8% (24.7%, 96.0%) and 47.0% (18.6%, 86.0%), respectively. Table 2 presents the MPR of OACs and anti-platelet agents during the follow-up period for both groups. Adherence to DOACs was better than adherence to warfarin (median MPR, 72.8% vs 47.0%). According to the MPR, a cross-over between DOACs and warfarin and the concomitant use of anti-platelet agents during follow-up were rare.

**Table 1:** Baseline characteristics of patients before and after inverse probability of treatment weighting according to the type of OAC.

	Before IPTW			After IPTW		
	DOAC (n = 106)	Warfarin (n = 319)	SMD	DOAC (n = 106)	Warfarin (n = 319)	SMD
Demographic characteristics						
Age (years)	67.8 ± 9.4	66.2 ± 10.7	0.162	67.5 ± 10.0	66.5 ± 10.5	0.093
Male (%)	67.9	65.9	0.038	67.3	66.0	0.028
Comorbid conditions (%)						
Hypertension	51.9	47.8	0.079	50.7	50.3	0.008
Diabetes mellitus	35.8	22.5	0.295	24.4	27.3	0.065
Dyslipidemia	84.0	71.2	0.311	71.9	74.6	0.061
Heart failure	35.8	40.9	0.107	43.0	40.6	0.050
Myocardial infarction	7.5	6.2	0.050	5.6	6.3	0.026
Systemic arterial embolism	5.7	8.1	0.098	7.3	7.4	0.002
Peripheral arterial disease	32.1	29.1	0.063	30.4	29.6	0.019
Chronic liver disease	50.9	44.1	0.135	49.6	46.5	0.062
Gastrointestinal bleeding	11.3	8.8	0.085	12.4	9.4	0.093
Unclassified major bleeding	10.4	8.8	0.054	8.8	9.1	0.013
CHA_2_DS_2_-VASc score	2.6 ± 1.4	2.5 ± 1.5	0.071	2.6 ± 1.5	2.6 ± 1.5	0.033
HAS-BLED score	3.3 ± 1.1	3.1 ± 1.2	0.127	3.3 ± 1.2	3.2 ± 1.2	0.117
Medication (%)						
DOAC	100.0	0.0	–	100.0	0.0	–
Dabigatran	2.8	0.0	–	2.4	0.0	–
Rivaroxaban	16.0	0.0	–	19.0	0.0	–
Apixaban	72.6	0.0	–	70.3	0.0	–
Edoxaban	8.5	0.0	–	8.3	0.0	–
Standard dose	10.4	–	–	9.4	–	–
Low dose	89.6	–	–	90.6	–	–
Warfarin	0.0	100.0	–	–	100.0	–
Anti-platelet agent						
Aspirin	27.4	19.1	0.196	21.7	21.0	0.017
Clopidogrel	22.6	13.8	0.240	15.3	15.3	0.002
Prasugrel/ticagrelor	0.0	0.0	–	0.0	0.0	–
Beta-blocker	34.9	26.9	0.173	31.7	28.8	0.063
ACEI/ARB	42.5	30.3	0.252	33.6	35.0	0.031
Calcium channel blocker	40.6	33.4	0.146	39.0	37.1	0.039
Loop diuretics	25.5	22.2	0.075	20.7	24.0	0.080
Statins	36.8	25.6	0.241	25.0	29.7	0.105
NSAID	45.3	46.9	0.035	51.7	48.0	0.074
Proton pump inhibitor	23.6	30.6	0.161	26.8	29.7	0.063

Data are expressed as % and mean ± SD.

*ACEI* angiotensin-converting enzyme inhibitor, *AF* atrial fibrillation, *ARB* angiotensin II receptor blocker, *DOAC* direct oral anticoagulant, *IPTW* inverse probability of treatment weighting, *OAC* oral anticoagulant, *SMD* standardized mean difference.

**Table 2:** Medication possession ratio before and after inverse probability of treatment weighting according to the type of OAC.

Medication possession ratio (%)	Before IPTW			After IPTW		
	DOAC (n = 106)	Warfarin (n = 319)	P value	DOAC (n = 106)	Warfarin (n = 319)	P value
OAC			0.004			0.008
DOAC	72.8 (24.7, 96.0)	0.0 (0.0, 0.0)		72.7 (24.7, 96.0)	0.0 (0.0, 0.0)	
Warfarin	0.0 (0.0, 0.0)	47.0 (18.6, 86.0)		0.0 (0.0, 0.0)	47.0 (18.6, 86.0)	
Aspirin	0.0 (0.0, 4.0)	0.0 (0.0, 4.0)	0.49	0.0 (0.0, 4.0)	0.0 (0.0, 4.0)	0.93
Clopidogrel	0.0 (0.0, 0.6)	0.0 (0.0, 0.4)	0.11	0.0 (0.0, 0.6)	0.0 (0.0, 0.4)	0.98
Prasugrel/Ticagrelor	0.0 (0.0, 0.0)	0.0 (0.0, 0.0)	0.32	0.0 (0.0, 0.0)	0.0 (0.0, 0.0)	0.32

Data are expressed as median (25th, 75th percentiles).

*DOAC* direct oral anticoagulant, *IPTW* inverse probability of treatment weighting, *OAC* oral anticoagulant.

### Clinical outcomes—DOAC vs warfarin

**Figure 2:**
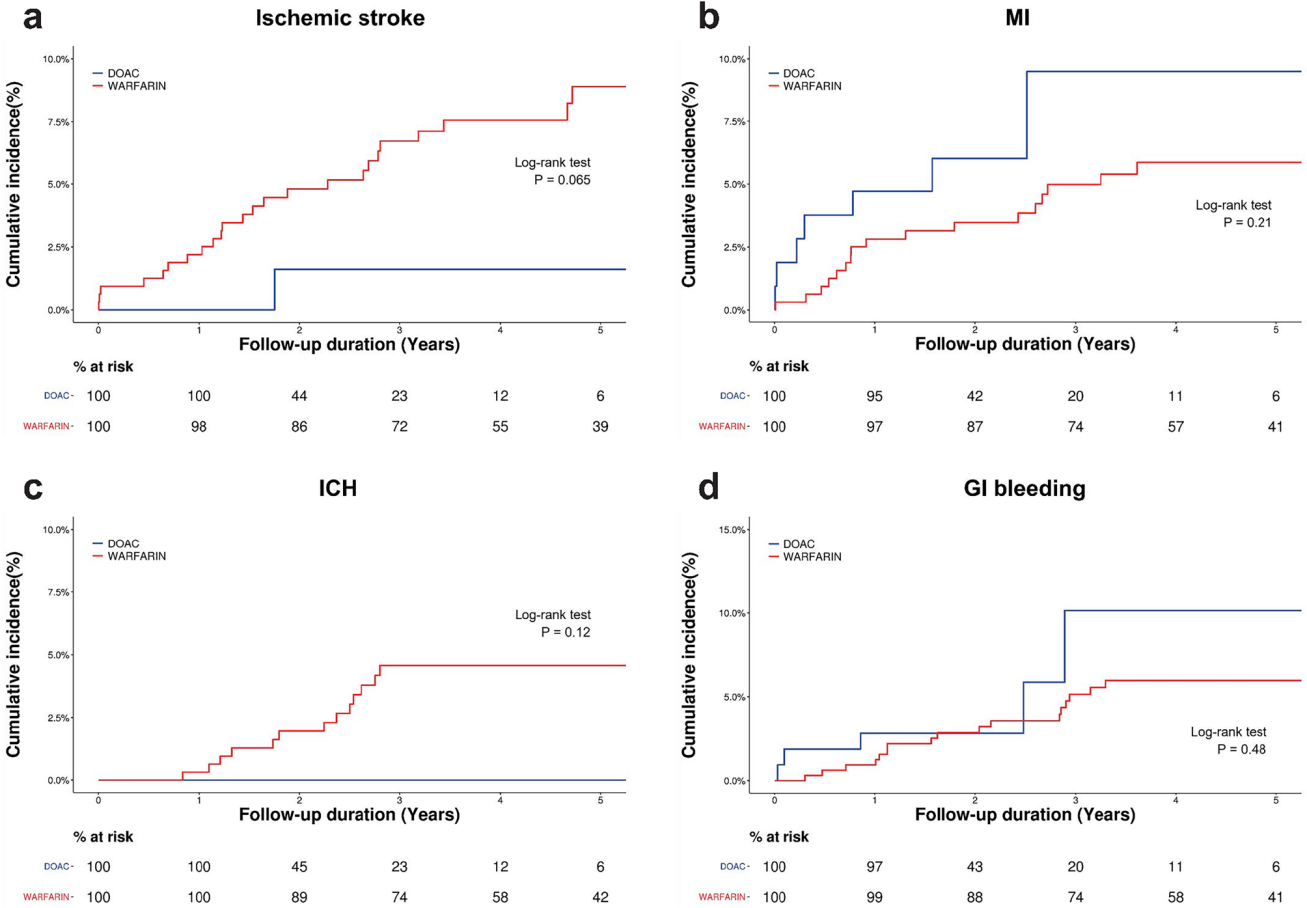
Unadjusted cumulative incidence rates for clinical outcomes in the DOAC and warfarin group—Cumulative incidence curves are shown for (**a**) ischemic stroke, (**b**) MI, (**c**) ICH, and (**d**) GI bleeding. All P values were calculated with the use of the log-rank test. *DOAC* direct oral anticoagulant, *GI* gastrointestinal, *ICH* intracranial hemorrhage, *MI* myocardial infarction.

During a median follow-up of 3.6 years (2.1 years, 5.5 years), the ischemic stroke, MI, ICH, and GI bleeding occurred in 1 (0.9%), 7 (6.6%), 0 (0.0%), and 5 (4.7%) in the DOAC group and 25 (7.8%), 17 (5.3%), 13 (4.1%), and 18 (5.6%) in the warfarin group, respectively. Figure 2 shows the unadjusted cumulative incidence rates for clinical outcomes of the DOAC and warfarin group.

After IPTW, the baseline characteristics were well-balanced between the two groups (all SMDs < 0.2) (Table 1). The weighted incidences of ischemic stroke, MI, ICH, and GI bleeding during a median follow-up of 3.2 years (1.8 years, 4.9 years) are presented in Table 3. Using an IPTW analysis, the weighted incidence of ischemic stroke was significantly lower in the DOAC group (HR 0.07, 95% CI 0.02 to 0.34, P = 0.001) compared to the warfarin group. However, the incidence of MI (HR 1.32, 95% CI 0.68 to 2.58, P = 0.41) and GI bleeding (HR 1.78, 95% CI 0.96 to 3.27, P = 0.06) were not significantly different between the two groups (Table 3). There was no ICH events in the DOAC group (therefore, we could not calculate the HR of ICH). However, the incidence rate was not significantly different between the two groups (P = 0.17).

**Table 3:** Risk of clinical outcomes in the DOAC and warfarin group with inverse probability of treatment weighting.

Clinical outcomes	Event rate per 100-person years		DOAC compared with warfarin	
	DOAC	Warfarin	Hazard ratio (95% CI)	P value
Ischemic stroke	0.18	1.76	0.07 (0.02–0.34)	0.001
Myocardial infarction	1.86	1.23	1.32 (0.68–2.58)	0.41
Intracranial hemorrhage	0.00	0.90	–	–
Gastrointestinal bleeding	2.65	1.31	1.78 (0.96–3.27)	0.06

*CI* confidence interval, *DOAC* direct oral anticoagulant.

## Discussion

This nationwide retrospective cohort study aimed to evaluate the effect of DOACs compared to warfarin on clinical outcomes in Asian patients with AF and ESRD undergoing chronic dialysis. Using NHI claims data in South Korea, present study yielded several major finding as follows: (1) Among patients with non-valvular AF and ESRD undergoing dialysis, a quarter of new OACs users were prescribed DOACs. Notably, the adherence to DOACs was better than to warfarin; (2) DOACs were associated with a reduced risk of ischemic stroke in Asian patients with AF and ESRD undergoing dialysis compared with warfarin over a median follow-up of 3.2 years; (3) Rates of MI, ICH, and GI bleeding were comparable between DOACs and warfarin users.

Although warfarin has been used mainly for patients with AF and ESRD undergoing dialysis considered eligible for OACs, previous meta-analysis showed that warfarin use was associated with no significant change for the risk of ischemic stroke and a significantly higher risk of hemorrhagic stroke compared to non-warfarin use in patients with AF undergoing dialysis^7^. Similar findings were observed in a Korean nationwide population-based study^17^. Beyond bleeding concerns, warfarin is associated with calcific uremic arteriopathy, a rare, serious complication, characterized by vascular medial calcification, thrombosis, and intense inflammation in patients with ESRD^18^. DOACs were shown to have a favorable risk–benefit profile compared to warfarin in patients with AF who do not have ESRD^3,4^. Furthermore, DOACs were more effective and safer in Asians than in non-Asians^19^. However, the use of DOACs for patients with AF and ESRD undergoing dialysis is controversial. The 2018 European guidelines recommend against DOAC use in dialysis^20^. Notably, DOACs have not been approved in Europe for patients undergoing dialysis. By contrast, the 2019 Focused Update of the 2014 AHA/ACC/HRS guideline recommended warfarin [international normalized ratio (INR) 2.0 to 3.0] or apixaban for patients with AF who have ESRD undergoing dialysis, with a class IIb recommendation^21^. In this study, we observed that a quarter of new OACs users in patients with non-valvular AF and ESRD undergoing dialysis were prescribed DOACs. This is similar to a previous study reporting that 26.6% of patients on newly anticoagulated hemodialysis were started on a DOACs^22^. About 90% of DOACs were apixaban and rivaroxaban in this study, which are less dependent on renal excretion (27% to 33%), and they were approved for use in ESRD in the United States.

Recently, 3 RCTs have compared DOACs with vitamin K antagonists (VKAs) in patients with AF on hemodialysis. The Valkyrie study compared VKA (n = 44) with 10 mg of rivaroxaban (n = 46) and 10 mg of rivaroxaban combined with vitamin K2 (n = 42) in 1:1:1 allocation ratio^23^. It showed that rivaroxaban with or without vitamin K2 reduced the primary endpoint (composite of fatal and non-fatal cardiovascular events) compared with VKA during a median follow-up of 1.9 years. Compared with the VKA arm, the risk-adjusted HR for the primary endpoint was 0.41 in the rivaroxaban (P = 0.0006) and 0.34 in the rivaroxaban with vitamin K2 arm (P = 0.0003). Although life-threatening and major bleeding occurred more often in the VKA arm than in the rivaroxaban arm, there was no difference between the treatment groups regarding all-cause death, cardiac death, or risk of stroke. After adjustment for competing risk of death, the HR for life-threatening and major bleeding of the pooled rivaroxaban arm was 0.44 compared with the VKA arm (P = 0.02). The Renal Hemodialysis Patients Allocated Apixaban Versus Warfarin in Atrial Fibrillation (RENAL-AF) study was a prospective, randomized, open-label, blinded-outcome evaluation of apixaban versus warfarin in patients receiving hemodialysis with AF and CHA_2_DS_2_-VASc score ≥ 2 during a median follow-up of 11 months^24^. Patients were randomly assigned 1:1 to apixaban 5 mg BID (2.5 mg BID for patients ≥ 80 years, weight ≤ 60 kg, or both) or dose-adjusted warfarin. It was terminated prematurely after randomizing 154 patients (82 patients assigned to the apixaban group and 72 patients assigned to the warfarin group) because of severe recruitment problems. Hence, there was inadequate power to draw any conclusion regarding the rates of major or clinically relevant non-major bleeding comparing apixaban and warfarin. The Compare Apixaban and Vitamin K Antagonists in Patients With Atrial Fibrillation and End-Stage Kidney Disease (AXADIA-AFNET 8) study was a prospective, randomized, open blinded end point trial comparing the effect of apixaban 2.5 mg BID (n = 48) to dose-adjusted VKA (n = 49) in patients with AF on chronic hemodialysis^25^. There were no apparent differences in safety and efficacy between the two groups. However, the pre-specified non-inferiority test requirements were unmet because of slow enrollment. Early termination of the above RCTs left unanswered questions about the relative efficacy and safety of DOAC and VKA.

In the present study, DOACs were associated with a 93% reduction in the risk of ischemic stroke compared to warfarin in patients with AF and ESRD undergoing dialysis. A previous study also showed that DOAC was associated with a 45% reduction in the risk of stroke or systemic embolism compared to warfarin in patients with AF and stage 4 or 5 chronic kidney disease or undergoing hemodialysis albeit the 95% CI crossed the line of unity (HR 0.55, 95% CI 0.27–1.10)^26^. On the contrary, the Valkyrie study did not show the reduction of ischemic stroke (P = 0.20)^23^. We speculated that the marked reduction of ischemic stroke for the DOAC group compared to the warfarin group in the present study might be explained by the poor adherence to warfarin. It might be caused by high INR, minor, or major hemorrhage^27^. Considering the median MPR (47.0%) for warfarin in the warfarin group, the time in therapeutic range (TTR) in the warfarin group was expected to be lower than TTR in the previous RCTs (median TTR; 44–50.7%)^23–25^. A previous retrospective study reported that patients undergoing dialysis had a TTR of 42.4%, while patients with estimated glomerular filtration rate 60–89 mL/min/1.73 m^2^ had a TTR of 60.1% despite comparable INR monitoring intensity^28^. Poor control of TTR in patients with AF and ESRD undergoing dialysis could mitigate the stroke prevention effect of warfarin in real-world practice^29^. In the present study, the incidence rate per 100-person years for ischemic stroke was 0.18 in the DOAC group and 1.76 in the warfarin group. It was lower than previous studies (0.85 to 6.67 in the DOAC group and 1.44 to 5.30 in the warfarin group)^26,30^. This may be explained by differences in the study population. Contrary to previous studies, our study excluded patients with a history of stroke and transient ischemic attack; therefore, the CHA_2_DS_2_-VASc score was low (mean; 2.6 points versus 4.5 points). Previous studies had reported that no significant differences in the rates of ICH, GI bleeding, and MI between DOACs and warfarin^26,30,31^. In alignment with these previous studies, we found that ICH, GI bleeding, and MI were comparable between DOACs and warfarin. However, there were no ICH events in the DOAC group in this study, although there was no statistically significant difference in the incidence rates between the two groups. OAC-related ICH is the most challenging complication in patients with AF and has been associated with higher mortality^32^. The incidence of ICH during warfarin treatment was fourfold higher in Asians than in White individuals^33^. The incidence of ICH with DOACs is significantly lower than that with warfarin in both Asians and non-Asians^34^. Interestingly, DOACs seems to have a greater relative risk reduction of ICH in Asians than non-Asians although these findings emanated from studies excluding patients with ESRD^19^. Considering this superiority of DOACs for ICH compared with warfarin, DOACs would confer greater benefit to Asian patients with ESRD undergoing dialysis. Pharmacokinetic data from the RENAL-AF study^24^ showed that the 12-h area under the curve (AUC0-12) for apixaban 5 mg BID in RENAL-AF did not differ from the AUC0-12 for patients with an estimated creatinine clearance (eCrCl) of 15–59 mL/min from the Apixaban for Reduction in Stroke and Other Thromboembolic Events in Atrial Fibrillation (ARISTOTLE trial)^12^. In addition, AUC0-12 for apixaban 2.5 mg BID in RENAL-AF did not differ from the AUC0-12 for patients with eCrCl of 15–89 mL/min from the ARISTOTLE trial. This finding also supports the use of DOACs in patients with ESRD undergoing dialysis. However, further large-scale clinical studies are required to confirm these findings.

### Study limitations

Present study had several limitations. First, it was an observational, retrospective study with inherent limitations of a non-randomized study. Second, dabigatran was included in this study. Dabigatran may be inappropriate for patients with ESRD undergoing dialysis because it is mainly eliminated by the kidney (80%) and is dialyzable. However, dabigatran accounted for less than 3% of DOAC in this study. Third, it was based on administrative data from the HIRA database. We lacked laboratory test results, such as INR, similar to previous studies that used administrative databases. Therefore, we could not evaluate the quality of warfarin treatment. Finally, the study population may have been too small to have sufficient statistical power for analysis. However, considering that none of the DOACs have been approved for patients with ESRD undergoing dialysis in South Korea, this study is the largest study comparing the effects of DOACs and warfarin in Korean patients with AF and ESRD undergoing dialysis.

## Conclusions

In this nationwide cohort study of OAC-naïve Asian patients with AF and ESRD undergoing dialysis, DOACs may be associated with a reduced risk of ischemic stroke compared to warfarin. In addition, the MI, ICH, and GI bleeding rates may be comparable between DOACs and warfarin. Our results suggest that DOACs can be associated with superior efficacy and comparable safety outcomes compared to warfarin in this population. Further large prospective randomized clinical trials are need to verify these findings.

### Supplementary Information

Supplementary Table S1.

## Data Availability

The NHI claims data in South Korea were analyzed in the present study. Any researchers can access NHI claims data with HIRA’s approval. The HIRA will grant access to these data upon request from qualified, interested researchers (http://opendata.hira.or.kr/home.do).
