# Supplementary material for: Direct oral anticoagulants for oral anticoagulants-naïve Asian patients with atrial fibrillation and end-stage renal disease undergoing dialysis
Source: Sci Rep. 2024 Jun 25;14:14679. doi: 10.1038/s41598-024-65541-z (PMC11199492; doi:10.1038/s41598-024-65541-z)
Supplement: Supplementary file 1 — Supplementary Table S1. [file 41598_2024_65541_MOESM1_ESM.docx]

**Direct Oral Anticoagulants for Oral Anticoagulants-Naïve Asian Patients with Atrial Fibrillation and End-stage Renal Disease Undergoing dialysis**

Jae-Hyung Roh, MD^1^, Yong-Giun Kim, MD^2,*^, Doyeon Kim, BS^3^, Sangwoo Park^2^, Kyung Sun Park, MD^4^, Minsu Kim, MD^1^, Ki Won Hwang, MD^5^, Wonmook Hwang, MD^1^, Gyung-Min Park, MD^2^, Jae-Hwan Lee, MD^1^

^1^Division of Cardiology, Chungnam National University Sejong Hospital, Chungnam National University School of Medicine, Sejong, Korea.

^2^Department of Cardiology, Ulsan University Hospital, University of Ulsan College of Medicine, Ulsan, Korea.

^3^Graduate School of Data Science, Kyungpook National University, Daegu, Korea.

^4^Department of Nephrology, Ulsan University Hospital, University of Ulsan College of Medicine, Ulsan, Korea.

^5^Division of Cardiology, Pusan National University Yangsan Hospital, Pusan National University of Medicine, Yangsan, Korea.

*Corresponding Author: Yong-Giun Kim, MD. E-mail: apollo0822@naver.com

**Supplementary Table S1.** Definitions of comorbidities, scores, and clinical outcomes

| **Diagnosis** | **ICD-10-CM code and definition** | **Diagnostic definition** |
| --- | --- | --- |
| **Inclusion/exclusion criteria** |  |  |
| **Atrial fibrillation** | I48 | Admission or outpatient department≥1 |
| **History of stroke, transient ischemic attack, and intracranial hemorrhage** | G45, G46, H34.0, I60-I69 |  |
| **Valvular atrial fibrillation** | I05.0, I05.2, I05.9, Z95.2-Z95.4 | Admission or outpatient department≥1 |
| **Comorbidities** |  |  |
| **Hypertension** | I10-I15; and minimum 1 prescription of anti-hypertensive drug (thiazide, loop diuretics, aldosterone antagonist, alpha-/beta-blocker, calcium-channel blocker, angiotensin-converting enzyme inhibitor, angiotensin II receptor blocker). | Admission≥1 or outpatient department≥2 |
| **Diabetes mellitus** | E10-E14; and minimum 1 prescription of anti-diabetic drugs (sulfonylureas, metformin, meglitinides, thiazolidinediones, dipeptidyl peptidase-4 inhibitors, α-glucosidase inhibitors and insulin). | Admission≥1 or outpatient department≥2 |
| **Dyslipidemia** | E78.0-E78.5 | Admission or outpatient department≥1 |
| **Heart failure** | I09.9, I11.0, I13.0, I13.2, I25.5, I42.0, I42.5-I42.9, I43, I50, P29.0 | Admission or outpatient department≥1 |
| **Myocardial infarction** | I21-I23 | Admission or outpatient department≥1 |
| **Systemic arterial embolism** | I74 | Admission or outpatient department≥1 |
| **Peripheral arterial disease** | I70, I71, I73.1, I73.8, I73.9, I77.1, I79.0, I79.2, K55.1, K55.8, K55.9, Z95.8, Z95.9 | Admission or outpatient department≥2 |
| **Chronic liver disease** | B15-B19, K70-K77, K80-87, I85, I86.4, I98.2, I98.3, Z94.4 | Admission or outpatient department≥1 |
| **Gastrointestinal bleeding** | K22.11, K22.6, K25.0, K25.2, K25.4, K25.6, K26.0, K26.2, K26.4, K26.6, K27.0, K27.2, K27.4, K27.6, K28.0, K28.2, K28.4, K28.6, K29.0, K31.81, K55.21, K57.01, K57.03, K57.11, K57.13, K57.21, K57.23, K57.31, K57.33, K57.41, K57.43, K57.51, K57.53, K57.81, K57.83, K57.91, K57.93, K62.5, K92.0-K92.2 | Admission or outpatient department≥1 |
| **Unclassified major bleeding** | D62, H05.2, H35.6, H43.1, J94.2, M25.0, R04 |  |
| **Scores** |  |  |
| **CHA_2_DS_2_-VASc score** | Heart failure (1 point), hypertension (1 point), age (65-74 years; 1 point, ≥75 years; 2 points), diabetes (1 point), previous stroke/systemic embolism/transient ischemic attack (2 points), vascular disease (prior MI or PAD, 1 point) and female sex (1 point) | |
|  |  |  |
| **Modified HAS-BLED score*** | Hypertension (1 point), liver disease (1 point), renal disease (1 point), stroke history (1 point), bleeding history (1 point), age>65 years (1 point) and drug (concomitant use of NSAID or antiplatelet agent, 1 point) | |
| **Clinical outcome** |  |  |
| **Ischemic stroke** | I63, I64 | Primary diagnosis, admission≥1, and brain imaging (CT or MRI) ≥1 |
| **Myocardial infarction** | I21-I23 | Primary diagnosis, admission≥1 and coronary angiography |
| **Intracranial hemorrhage** | I60-62 | Primary diagnosis, and admission≥1 |
| **Gastrointestinal bleeding** | K22.11, K22.6, K25.0, K25.2, K25.4, K25.6, K26.0, K26.2, K26.4, K26.6, K27.0, K27.2, K27.4, K27.6, K28.0, K28.2, K28.4, K28.6, K29.0, K31.81, K55.21, K57.01, K57.03, K57.11, K57.13, K57.21, K57.23, K57.31, K57.33, K57.41, K57.43, K57.51, K57.53, K57.81, K57.83, K57.91, K57.93, K62.5, K92.0-K92.2 | Primary diagnosis, and admission≥1 |

CT, computed tomography; MRI, magnetic resonance image.
